# Coccidia Vaccine Challenge and Exogenous Enzyme Supplementation in Broiler Chicken 1. Effect on Digesta Viscosity, Diet Energy Utilization, and Apparent Metabolizable Energy Value of Wheat

**DOI:** 10.3390/ani11030641

**Published:** 2021-02-28

**Authors:** Andrew Dunaway, Sunday A. Adedokun

**Affiliations:** Department of Animal and Food Sciences, University of Kentucky, Lexington, KY 40546, USA; 1992ad@gmail.com

**Keywords:** coccidiosis, metabolizable energy, broiler chicken, corn, wheat, enzyme

## Abstract

**Simple Summary:**

*Eimeria* species parasite can have great impacts on the health and nutrient and energy utilization of broiler chickens. This study examined the effect of *Eimeria* species challenge on broiler chickens’ energy utilization when fed two types of diets with or without exogenous enzyme supplementation. Seven-day post-challenge (day 21), wheat apparent metabolizable energy was lower in birds challenged with coccidia vaccine. By day 28 (14-day post-challenge), there was no difference in the apparent metabolizable energy value of wheat. The addition of exogenous enzyme resulted in an improvement in nitrogen utilization (~6%) in challenged birds fed the corn-SBM-based diets seven-day post-challenge. This study showed that broiler chickens were negatively impacted by the coccidia vaccine challenge seven-day post-challenge, but were able to compensate for the reduction in apparent metabolizable energy of wheat 14-day post-challenge. Furthermore, enzyme supplementation decreased the digesta viscosity of birds fed a wheat-corn-soybean meal-based diet 14-day post challenge.

**Abstract:**

This study examined the effect of exogenous mixed-enzyme supplementation (xylanase, β-glucanase, and pectinase) to a corn-SBM (CS) and a wheat-CS-based (WCS) diet in birds challenged with coccidia vaccine (Coccivac B-52™). The WCS-based diet was produced by replacing 30% of the energy-yielding portions of the CS-based diet with wheat. On day 14, 448 (*n* = 7) Cobb by-product breeder male broilers were assigned to a 2 (diet types) × 2 (with or without enzyme supplementation) × 2 (0 or 20 × coccidia vaccine challenge; CVC) factorial arrangement of treatments in a completely randomized design for the determination of the apparent metabolizable energy (AME) value of wheat. Treatment effects on jejunum digesta viscosity and AME corrected for nitrogen (AMEn) of the diets were evaluated within each diet type as a 2 × 2 factorial arrangement of treatments, 7- and 14-day post-challenge. Seven-day post-challenge (day 21), dry matter (DM) and energy utilization, AME, and AMEn of the CS- and WCS-based diets decreased (*p* < 0.05) with CVC. Both AME and AMEn of wheat decreased (*p* < 0.05) by about a 20% in CVC-birds 7-day post-challenge. Enzyme and CVC resulted in a decrease (*p* < 0.05) in jejunal digesta viscosity in birds fed the CS-based diets, while there was an interaction (*p* < 0.05) between CVC and enzyme, with enzyme lowering (*p* < 0.05) the viscosity of digesta 7-day post-challenge. Results from this study showed that CVC resulted in a 20% decrease in AMEn 7-day post-challenge, while the interaction between exogenous enzyme supplementation and CVC resulted in an improvement in nitrogen utilization (~6%) in CVC birds fed the corn-SBM-based diet 7-days post challenge.

## 1. Introduction

In 2019, there were 43.9 billion pounds of broiler chickens produced, up from 35.5 billion a decade earlier [1]. Due to the demand for broiler-meat production, the amount of feed needed for production will continue to increase. Feed costs account for more than 60% of the costs involved in poultry production [2], and the majority of feed costs come from the energy-containing ingredients. Due to this, it is important to have access to updated energy values of the various feed ingredients used in poultry feed, to better meet the requirements of the birds and reduce feed wastage through overfeeding.

In addition to feed costs, infection from the protozoan (*Eimeria* species) that causes coccidiosis has had major impacts on commercial poultry production. Broiler chickens are affected by the *Eimeria* family of parasitic protozoan pathogen, which can increase mortality and morbidity in the birds with the clinical form of infection [3]. In both the clinical and sub-clinical forms of coccidiosis, birds may show reduced performance, such as reduced feed intake and body weight gain. They may also show reduced nutrient and energy retention, leading to reduced apparent metabolizable energy (AME) from the diet [4,5]. In both cases, there are major economic losses, of which the annual global costs to poultry production have been estimated to be over $2.2 billion [6].

Soluble non-starch polysaccharides (NSP) are found in plant-based feed ingredients, and are known to possess antinutritive effects, such as increased digesta viscosity, decreased performance, reduced AME retention, reduced villi size in the small intestine, and sticky droppings [7,8,9,10,11,12,13]. Corn and wheat are two common energy-containing feed ingredients used in broiler production. Wheat tends to have higher levels of soluble NSP and that may negatively affect the bird’s ability to utilize nutrients and energy in their diet. Carbohydrase enzymes, specifically NSPase, may be supplemented to the diet to counteract some of the antinutritive effects of NSP. There is evidence that soluble NSP can increase Gram-negative bacteria (i.e., *Escherichia coli*) and decrease Gram-positive bacteria (i.e., lactic acid-producing bacteria), but reducing the viscosity through enzyme supplementation may promote an environment less suited for Gram-negative bacterial proliferation [12].

The fact is, when birds are under coccidia vaccine challenge (CVC) and given a diet high in soluble NSP, both of these may affect the birds’ ability to sequester energy, thereby reducing the AME retention value of the diet or ingredient. Whereas, exogenous enzyme supplementation may improve the nutrient and energy utilization of the diet. Thus, the objectives of this study were to compare the effect of CVC and exogenous enzyme supplementation on AME and AMEn values of CS- and WCS-based diets and wheat in broiler chickens, 7- (day 21; peak-CVC) and 14- (day 28; recovery phase) day post-CVC.

## 2. Materials and Methods

The management of the birds, experimental procedures, and sample collections for the experiment followed the standard operating procedures for the animal facility, and was reviewed and approved by the University of Kentucky Animal Care and Use Committee (Protocol # 2018-2890).

### 2.1. Birds and Diets

The experiment used day-old by-product breeder chicks obtained from Cobb Monticello, KY. On day zero, 500 day-old broiler chicks were individually tagged and fed a standard corn-SBM-based broiler starter diet that met or exceeded nutrient and energy requirements from day 0 to 14. Birds were raised in battery cages in an environmentally controlled room with 20 h of light and 4 h of dark. All birds had unrestricted access to feed and water throughout the duration of the experiment. A total of 448 birds were individually weighed and randomized to treatments on day 14 in a completely randomized design. Experimental treatments were arranged in a 2 × 2 × 2 factorial, for a total of eight treatments and seven replicates/treatment, with eight birds per replicate cage. Four birds/cage were sampled on days 21 (the two heaviest and two lightest birds) while the remaining four birds/pen were sampled on day 28. All birds were weighed and euthanized by CO_2_ asphyxiation prior to sampling on days 21 and 28.

### 2.2. Coccidia Vaccine Challenge

The reference diet used was a corn-SBM-based diet (CS) in which 30% of the energy yielding portion of the diet (corn, SMB, and soy oil; Table 1) was replaced with wheat to produce the wheat-corn-SBM-based diet (WCS). Similar ratios of the energy yielding components of the diets were maintained across all the diets used in this study. The exogenous enzyme containing diets were produced from the same basal diet by supplementing a multi-carbohydrase enzyme (Ronozyme^®^ WX2 and Ronozyme^®^ VP, DSM Animal Nutrition, Parsippany, NJ, USA) to both the CS and WCS diets (as a ground-corn-based premix, Table 1). Ronozyme^®^ WX2 (xylanase) premix was added to the diet to supply 0.1 g/kg of the enzyme/kg of feed, while Ronozyme^®^ VP (glucanase + pectinase) premix was added to the diet to supply 0.25 g of the enzyme/kg of the diet, as per the manufacturer’s recommendation (DSM, Parsippany, NJ, USA). The diets with enzyme supplementation were produced from the same basal diet as diets without enzyme supplementation.

Birds in the non-CVC treatments were orally gavaged on day 14 with 0.6 mL of distilled water, whereas CVC birds were orally gavaged with 20× of coccidia vaccine (Coccivac^®^-B52, Merck Animal Health, Omaha, NE, USA) in 0.6 mL distilled water. The vaccine contained live *Eimeria* occysts of *E. acervulina, E. maxima, E. mivati, and E. tenella*. The total collection method was used to determine energy and nitrogen retention, as well as the AME and AME corrected for nitrogen (AMEn). Seventy-two hours before each sampling on day 21 and 28, the excreta collection trays were cleaned, the feed was removed from the feeders and weighed at 0 and 72 h. On days 19, 20, 21, and 26, 27, 28 excreta was quantitatively collected and weighed each morning at the same time before storing at −20 °C, prior to drying in a forced-air oven at 55 °C for six days. Dried excreta samples were weighed and pooled by cage. Dried excreta samples, ingredients (corn, wheat, and SBM), and diets were ground to pass through a 0.5 mm screen using a mill grinder (Wiley Mill Standard Model No. 3, Arthur H. Thomas Co., Philadelphia, PA, USA).

### 2.3. Histological Analysis

The middle section of the ileum (about an inch) was taken on day 21 to determine the morphometric changes associated with the different treatments, especially the effect of *Eimeria* species on the ileum. The CVC used in this study is capable of affecting the entire gastrointestinal tract. Sample was taken from the ileum to specifically confirm the effects of the challenge on the gastrointestinal tract of the birds. Samples were processed (stained with haematoxylin and eosin) at the University of Kentucky’s Animal Diagnostics Lab (ADL). Ten villi height and crypt depth measurements were made per slide at 10**×** (upright clinical microscope, Model Eclipse Ci-E, Nikon Corporation, Tokyo, Japan). Villi height to crypt depth ratio (VHCD) was also determined.

### 2.4. Viscosity

Jejunal digesta was taken from the two heaviest birds (to have adequate sample quantities)/cage on days 21 and 28 and stored at −20 °C prior to the determination of the digesta viscosity. Digesta from the jejunum was taken for viscosity measurements, because this is the section of the mid-gut where considerable digestion and absorption take place. Approximately 2 g of thawed digesta were centrifuged (11.500× *g* for 15 min at 20 °C) and the viscosity was determined on 0.5 mL of supernatant using a viscometer (Vibro viscometer, model SV-1 A, A&D Company Limited, Tokyo, Japan) at 40 °C (body temperature of chickens).

### 2.5. Chemical Analysis

Diets and excreta samples were analyzed for dry matter (DM), gross energy (GE), and nitrogen. The DM (method 934.01) contents of the samples (feed ingredients, diets, and excreta) were determined by drying the samples at 110 °C for 16 h [14]. Nitrogen (method 990.03) contents of the samples were determined by the combustion method (model FP2000, Leco Corp., St. Joseph, MI, USA) [15], with EDTA as the internal standard. The GE of the feed ingredients, diets, and excreta samples was analyzed using a bomb calorimeter (Parr adiabatic bomb calorimeter, model 6200, parr instruments, Moline, IL, USA) with benzoic acid as the calibration standard. Feed ingredients were sent to the University of Missouri for proximate composition value determination as shown in Table 2.

The moisture, crude fat, crude fiber, and ash of corn, wheat, and soybean meal were determined at the University of Missouri Agriculture Experiment Station Chemical Laboratories (Columbia, MO, USA). Crude fat (method 920.39) was determined by ether extraction [14]. Crude fiber (method 978.10) content was also determined [14]. The ash (method 942.05) contents of the feed ingredients were also determined [14]. Proximate analyses of ingredients are contained in Table 2.

### 2.6. Calculations and Statistical Analysis

The coefficient of energy and N retention was determined using the equation:

(1) $$Retention\;\left(\%\right)=\left(\frac{Cinput-Coutput}{Cinput}\right)\times 100$$

where C is the component being measured (i.e., energy and N). 

Apparent metabolizable energy was calculated using the following equation:

(2) AME=GE×cEM

where GE is the gross energy of the diet and cEM is the coefficient of energy metabolizability (cEM). 

The cEM of the test feed ingredient (wheat) was calculated using the indirect method after correcting for the non-energy yielding portions of the diets [16].

(3) $$EMti=EMtd-\frac{EMrd\times \left(1-FCti/td\right)}{FCti/td}$$

where EMti is the cEM of the test ingredient, EMtd is the cEM of the test diet, EMrd is the cEM of the reference diet, and FCti/td is the fractional contribution of the test ingredient to the test diet. The caloric value of 8.22 kcal/g was used to correct AME for N to give AMEn [17].

Data were analyzed using the GLM procedure of SAS (SAS Inst. Inc. Cary, Cary, NC, 2006). In order to take into consideration the nature of the two groups of the experimental diets (not isonitrogenous), the effects of CVC and enzyme supplementation on DM, N, energy utilization, AME, AMEn, digesta viscosity, and the histology of birds fed CS- and WCS-based diets were evaluated (within each diet group) as a 2 (non-CVC or CVC) × 2 (with or without exogenous enzyme supplementation) factorial arrangement of treatments. The test feed ingredient’s (wheat) AME and AMEn were analyzed as a 2 (non-CVC or CVC) × 2 (with or without exogenous enzyme supplementation) factorial arrangement of treatments. A cage served as the experimental unit, except for jejunal viscosity (two birds/cage) and for histology (one bird/cage), and the number of replicates was seven per treatment, unless otherwise stated. Outliers (data outside mean ± 3 SD) were removed from the data prior to statistical analysis. Where necessary, mean separation was by Tukey’s test and the level of significance was set at *p* < 0.05. Main and simple effects data were presented.

## 3. Results

The analyzed (enzyme analyses were done by DSM Nutritional Products, Parsippany NJ, USA) levels of the individual enzyme activities in the control diets were not higher than 5.0 FBG/kg (3.2 and 5.0 FBG/kg, for corn- and wheat-based diets, respectively) for glucanase while xylanase level was below the detection limit. The level of glucanase (from Ronozyme^®^ VP) was 18.4 FBG/kg while the level of xylanase (from Ronozyme^®^ WX2) was 259 FXU/kg for the corn-SBM-based diet. The corresponding levels of glucanase and xylanase in the wheat-corn-SBM-based diet were 24.5 FBG/kg and 311 FXU/kg, respectively.

### 3.1. Nutrient and Energy Retention

Significant two-way interaction was observed for nitrogen utilization with CVC without enzyme supplementation resulting in the lowest (*p* < 0.05) value, however, enzyme supplementation to the CS-based diets fed to CVC birds resulted in a 6% increase (*p* < 0.05) in nitrogen utilization compared to the supplemented CS-based diets (Table 3). Dry matter and energy utilization, AME, and AMEn values from birds fed the CS- and WCS-based diets were reduced (*p* < 0.05) by CVC 7-days post-challenge (Table 3 and Table 4). By day 28 (14-days post-challenge), the effects of CVC and enzyme supplementation on dry matter, nitrogen, energy utilization, AME, and AMEn were not significant (Table 5 and Table 6).

### 3.2. AME Contents of Diets and Wheat

The AME (19.6%) and AM En (20.8%) values of wheat were reduced with CVC 7-days post challenge (Table 7), while the effect of CVC and enzyme supplementation on wheat AME and AMEn was not significant 14-days post-challenge (Table 7).

### 3.3. Viscosity and Ileal Histology

Coccidia vaccine challenge and enzyme supplementation reduced (*p* < 0.05) jejunal digesta viscosity in birds fed the CS-based diets. Significant two-way interaction between CVC and enzyme supplementation was observed for jejunal digesta viscosity in birds fed WCS-based diets 14-day post-challenge (Table 8). Enzyme supplementation reduced (*p* < 0.05) digesta viscosity in both the unchallenged and CVC birds whose diets with the lowest (*p* < 0.05) viscosity observed in the CVC birds whose diets were supplemented with enzymes. The viscosity between the unchallenged birds fed diets containing enzyme supplementation and the CVC birds without enzyme supplementation was not different (14-day post-challenge; Table 8). Fourteen-day post-challenge, CVC and enzyme supplementation reduced (*p* < 0.05) jejunal digesta viscosity in birds fed WCS-based diets (Table 8).

In birds fed CS- and WCS-based diets, the VH and VH:CD decreased, while CD increased with CVC 7-day post-challenge (Table 9). In birds fed WCS-based diets, VH:CP was highest (*p* < 0.05) in the non-CVC birds whose diet was supplemented with enzyme 7-day post-challenge (Table 9).

## 4. Discussion

The demand for chicken meat will continue to grow, and the need for accurate AMEn of feed ingredients under different production conditions is pressing. While coccidiosis infection still plagues poultry producers, with increased bird mortality and decreased performance, determining the nutrient and energy retention of different feed ingredients in coccidia-challenged birds can further our understanding of how birds utilize different feed ingredients. Different energy-yielding feed ingredients fed to broiler chickens have inherently different properties. There are obvious differences in nutrient and energy values, however there are also physicochemical properties that may change how the birds utilize the nutrients and energy provided by the diet. Wheat contains higher levels of soluble NSP than corn, which has been shown to decrease AMEn and have other antinutritive effects, along with other common feed ingredients (e.g., barley, rye, and triticale) used in poultry production [11,18]. Enzyme supplementation has been shown to reduce some of the antinutritive effects of soluble NSP [9,19], which may improve the birds’ ability to utilize ingredients high in soluble NSP. Through the various ways AMEn can be reduced or improved, a deeper look into individual ingredients could prove beneficial to the costs associated with feeding broiler chickens.

During the peak of CVC infection (7-day post-challenge; day 21), the two-way interaction of N retention was significantly lower in CVC birds fed the CS-based diets with no enzyme supplementation. Although the effect of exogenous enzyme supplementation to the diets of the unchallenged birds was not significant, exogenous enzyme supplementation improved N retention in CVC birds by 6%. Furthermore, DM and energy utilization, AME, and AMEn were all reduced 7-day post CVC. This observation for CVC birds was an expected result of the challenge and follows in line with the energy retention on day 21. The reduction in the determined energy retention, AME, and AMEn values in the CVC birds showed that the challenge affected the birds’ ability to utilize energy from the diets. Several studies have reported some adverse effects of *Eimeria* sp. challenge on the ability of broilers to optimally utilize energy and nutrients in their feed [20,21]. These adverse effects include the destruction of intestinal villi, and thickening and shortening of the villi [22], which leads to a reduction in the surface area available for absorption, as well as a prioritization of gut development, as shown by increased crypt depth [23]. For the WCS-based diets, the effect of CVC on the birds 7-day post-challenge was similar to what was obtained for birds on the CS-based diets. For both the CS- and WCS-based diets, by day 28 (14-day post-challenge), the birds’ determined energy retention, AME, and AMEn values of the diets were no longer affected by CVC, therefore the pathogenicity of the coccidia infection had decreased, and the birds were already at the recovery phase. It is important to note that unlike under a controlled experimental condition, commercial broiler chickens are raised on the floor, which enables them to be able to recycle oocysts from the litter resulting in sustained compromise to the gastrointestinal tract. Furthermore, the compromised digestive tract presents a perfect environment for secondary bacterial infection to occur [24,25,26].

The determined values of wheat AME and AMEn through the difference method 7-day post-challenge (day 21) for CVC-birds fed CS-based diet resulted in a ~20% decrease in AME (3 379.8 kcal/kg vs 2 718.3 kcal/kg) and AMEn (3 296.6 kcal/kg vs 2 609.6 kcal/kg) when compared with that of the non-CVC birds. This observation, again, confirms the effect that CVC had on the birds’ absorptive capabilities through the damage caused by the infection to the epithelial lining of the small intestine. By day 28 of the study (14-day post-challenge), unlike the significant reduction in wheat’s AME and AMEn, there were no differences in the AME and AMEn of wheat between the CVC and non-CVS birds. This showed that the effects of CVC had significantly reduced, and the birds were able to better utilize the energy in the wheat. The addition of exogenous enzymes to the diet did not improve the AME and AMEn values. One of the reasons for this observation could be because the diets were not formulated to be deficient in energy therefore, may not have revealed the potential benefits to exogenous enzyme supplementation.

The AME and AMEn of wheat determined in the non-CVC birds without exogenous enzyme supplementation were 3368.9 kcal/kg and 3290.4 kcal/kg, respectively, 7-day post challenge and 3438 and 3353, respectively, 14-day post-challenge. The same treatment group on day 28 was similar for AME and AMEn with a slight increase of around 60 kcal/kg. The reported AMEn of hard red wheat is 2900 kcal/kg [18]. The determined AMEn values for 21 and 28 day were around 400 kcal/kg higher than those reported by [18], however a study using birds of similar age determined the AMEn of wheat to be 3372 kcal/kg using the regression method [27]. The relatively higher AME values could be due to differences in the cultivar (or improvement in seed breeding) of wheat used or an improvement in the efficiency with which modern day broilers are able to utilize feed ingredients.

Jejunal digesta viscosity of birds fed CS-based diets decreased by 11% as a result of CVC, while it decreased by 5% as a result of enzyme supplementation. For birds on WCS-based diets, interaction between CVC and enzyme supplementation resulted in non-CVC birds fed diets without enzyme supplementation having the highest viscosity, while the combination on CVC and enzyme supplementation resulted in the lowest viscosity. This was expected because both the CVC and enzyme supplementation individually reduced jejunal viscosity. Irrespective of the state of challenge (CVC or non-CVC), enzyme supplementation significantly reduced jejunal digesta viscosity. Lazaro [28] showed a similar trend in the decreased viscosity through exogenous supplementation of a carbohydrase blend in the diet. Interestingly, the significant effect of CVC and enzyme supplementation in reducing digesta viscosity in birds on CS-based diets 7-day post-challenge disappeared 14-day post challenge. However, this effect persisted in birds on WCS-based diets 14-day post challenge, in which case, CVC and enzyme supplementation reduced jejunal digesta viscosity by 8 and 16%, respectively. This reduction in jejunal digesta viscosity indicates that during the recovery phase (14-day post-CVC), enzyme supplementation to the WCS-based diets further reduced digesta viscosity (16% vs. about 0% for CS-based diet). The interaction between CVC and enzyme showed that the supplementation of exogenous enzymes significantly reduced jejunal digesta viscosity (7-day post- challenge) when added to a WCS-based diet, but no interaction was observed when for both CS- and WCS-based diets 14-day post challenge. The presence of an appropriate substrate is key to the effect of enzyme supplementation in this study. This is evidence for the efficacy of the Ronozyme^®^ enzyme in reducing viscosity in diets high in NSP. Lazaro [28] reported a similar trend in the decreased viscosity through exogenous supplementation of a carbohydrase blend in the diet. Exogenous xylanase supplementation was also shown to significantly reduce jejunal and ileal digesta viscosity in broilers fed wheat-based diets [29,30].

For birds on a CS-based diet 7-day post-CVC, no significant interaction between CVC and enzyme supplementation was observed for villi height, crypt depth, and the ratio between villi height and crypt depth. Despite the lack of significant interaction, CVC resulted in shorter villi, deeper crypt depth, and smaller villi height to crypt depth ratio. Similar trends were observed for the villi height and crypt depth of CVC-birds fed a WCS-based diet. Based on this observation, it is clear that the *Eimeria* infection led to significant damage of the villi, and thus leading to decreased surface area for absorption. The two *Eimeria* species that target the ileum of chickens contained in the Coccivac^®^-B52 used in this study are *E. acervulina* and *E. maxima*, with *E. maxima* being the most pathogenic of the two [3]. Although the data are not presented, we know that *E. acervulina* and *E. mivati* are found to infect the duodenum, and *E. maxima* also infects the jejunum of the SI [31], which may have further led to reduced nutrient and energy utilization [10]. At 7-day post challenge for bids fed a WCS-based diet, ileal VH:CD showed a two-way interaction. There was no difference in VH:CD for CVC birds, with or without enzyme supplementation, and non-CVC birds without enzyme supplementation. However, enzyme supplementation to the WCS-based diet in the absence of CVC resulted in higher VH:CD compared to the other treatments. This increase was driven mostly by a reduction in crypt depth (118.5 vs average of 168 µm). This increase in VH:CD as a result of enzyme supplementation would suggest that the enzymes increased the ileum’s surface area for absorption in the small intestine. Despite this observation, performance parameters, nutrient and energy retention, AME, and AMEn did not significantly increase with the increased absorptive capabilities in the ileum during the two-week period of this study. Future studies may explore the long-term effects of CVC on performance when birds are fed a WCS-based diet without mixed carbohydrase enzyme supplementation.

## 5. Conclusions

In most cases, the birds met the expectations of this study. The coccidia infection clearly affected the birds’ nutrient and energy retention, AME, and AMEn 7-day post challenge, but by 14-day post-CVC, the treatment effects disappeared. The lack of difference in AME and AMEn values 14-day post-challenge could be attributed to the waning effect of the CVC, because birds were already in the recovery phase because of the birds’ inability to recycle *Eimeria* oocyst from their excreta. Wheat AMEn 7-day post challenge was 20% higher in the non-CVC birds compared to the CVC birds (3297 vs 2610 kcal/kg) while the effect of CVC on wheat AME and AMEn was not significant 14-day post-challenge. Both the CVC and enzyme supplementation decreased digesta viscosity, but the decrease in digesta viscosity with enzyme supplementation for the WCS-based diet was higher compared to that of the CS-based diet (17 vs. 5%). The supplementation of glucanase, xylanase, and pectinase did not provide evidence of reducing the damage caused by CVC to the epithelial of the ileum. However, data from this study suggests that a CS-based diet may be better suited for CVC birds than a WCS-based diet. Future studies may look into long-term effects of CVC on performance when birds are fed a WCS-based diet without mixed carbohydrase enzyme supplementation.

## Figures and Tables

**Table 1:** Ingredient composition and analyzed dry matter, gross energy, and crude protein values of the experimental diets ^1^.

Ingredients, g/kg (As-Fed)	Without Enzymes		With Enzymes	
	CS	WCS	CS	WCS
Corn	639.6	438.6	619.6	418.6
Soybean meal, 48% CP ^2^	285.0	195.5	285.0	195.5
Soy oil	30.0	20.5	30.0	20.5
Wheat (hard red)	0.0	300.0	0.0	300.0
Dicalcium phosphate	17.6	17.6	17.6	17.6
Limestone	10.5	10.5	10.5	10.5
Vitamin-mineral premix ^3^	2.5	2.5	2.5	2.5
Salt	4.1	4.1	4.1	4.1
DL-methionine	3.0	3.0	3.0	3.0
L-lysine HCl	2.0	2.0	2.0	2.0
L-threonine	0.7	0.7	0.7	0.7
Titanium dioxide	5.0	5.0	5.0	5.0
Ronozyme^®^ WX2 premix ^4^	0.0	0.0	10.0	10.0
Ronozyme^®^ VP premix ^5^	0.0	0.0	10.0	10.0
Total	1000	1000	1000	1000
Analyzed nutrient and energy composition ^6^				
Gross energy, kcal/kg	4016.3	3918.4		
Dry matter, g/kg	895.0	889.5		
Crude protein (N × 6.25), g/kg	197.2	174.7		
Calcium, g/kg	9.4	9.2		
Phosphorus, g/kg	7.1	6.9		

^1^ CS = corn-SBM; WCS = wheat-corn-SBM; ^2^ CP = crude protein; ^3^ Vitamin-mineral premix was formulated to supply the following at 2.5 g per kilogram of diet: 11 025 IU of vitamin A; 3 528 IU of vitamin D; 33 IU of vitamin E; 0.91 mg of vitamin K; 2.21 mg of thiamin; 7.72 mg of riboflavin; 55 mg of niacin; 18 mg of pantothenate; 5 mg of vitamin B-6; 0.22 mg d-biotin; 1.10 mg of folic acid; 478 mg of choline; 0.03 of vitamin B-12; 75 mg of Zn; 40 mg of Fe; 64 mg of Mn; 10 mg of Cu; 1.85 mg of I; and 0.30 mg of Se; ^4^ The premix (ground corn-based) supplied 0.1 g of Ronozyme^®^ WX2 /kg of diet. The enzyme was added at the expense of ground corn; ^5^ The premix (ground corn-based) supplied 0.25 g of Ronozyme^®^ VP /kg of diet. The enzyme was added at the expense of ground corn; ^6^ The diets with enzyme supplementation were produced from the same basal diet as diets without enzyme supplementation.

**Table 2:** Analyzed proximate composition of the major energy yielding feed ingredients contained in the experimental diets (on as-is basis).

Component, g/kg	Corn	Wheat	Soybean Meal
Moisture	116.9	120.6	91.7
Gross energy, kcal/kg	3890.3	3873.3	4223.7
Crude protein (nitrogen × 6.25).	75.5	138.6	480.1
Crude fat	22.2	6.0	11.7
Crude fiber	16.5	21.8	31.2
Ash	13.3	16.1	62.2

**Table 3:** Main and simple effects of coccidia vaccine challenge (CVC) and exogenous enzyme supplementation on nutrient and energy retention, apparent metabolizable energy (AME), and apparent metabolizable energy corrected for nitrogen (AMEn) in 21-day-old broiler chickens fed corn-soybean meal-based diets (7-day post-challenge) ^1^.

Coccidia Vaccine Challenge	Enzyme	Dry Matter, %	Nitrogen, %	Energy, %	AME	AMEn
		**Means for Main Effect of CVC**				
−		73.0	70.1	75.9	3404	3318
+		62.2	63.0	62.4	2800	2692
		**Means for Main Effect of Enzyme**				
	−	66.9	65.8	68.4	3077	2979
	+	68.2	67.3	69.8	3126	3032
		**CVC × Enzyme**				
−	−	73.2	70.5 ^a^	76.0	3418	3333
+	−	60.7	61.2 ^c^	60.9	2737	2624
−	+	72.8	69.7 ^a^	75.7	3391	3303
+	+	63.6	64.9 ^b^	63.9	2862	2760
		
	SD ^2^	2.61	1.89	2.17	97.34	101.64
		**Probability**				
	CVC	<0.001	<0.001	<0.001	<0.001	<0.001
	Enzyme	0.251	0.076	0.146	0.236	0.219
	Enzyme × CCV	0.146	0.007	0.075	0.071	0.061

^1^ n for simple effects of minus CVC × minus Enzyme = 6, plus CVC × minus Enzyme = 6, minus CVC × plus Enzyme = 7, plus CVC × plus Enzyme = 6; ^2^ Standard deviation. ^a–c^ Means within a column without a common superscript differ (*p* < 0.05).

**Table 4:** Main and simple effects of coccidia vaccine challenge (CVC) and exogenous enzyme supplementation on nutrient and energy retention, apparent metabolizable energy (AME), and apparent metabolizable energy corrected for nitrogen (AMEn) in 21-day-old broiler chickens fed wheat-corn-soybean meal-based diets (7-day post-challenge) ^1^.

Coccidia Vaccine Challenge	Enzyme	Dry Matter, %	Nitrogen, %	Energy, %	AME	AMEn
		**Means for Main Effect of CVC**				
−		74.6	68.4	76.5	3371	3289
+		63.8	59.7	63.4	2792	2688
		**Means for Main Effect of Enzyme**				
	−	69.2	64.4	70.2	3095	3003
	+	69.2	63.7	69.7	3068	2973
		**CVC × Enzyme**				
−	−	74.2	68.5	76.3	3362	3281
+	−	64.1	60.2	64.2	2828	2726
−	+	75.0	68.4	76.8	3379	3297
+	+	63.4	59.1	62.6	2756	2650
	SD ^2^	2.24	2.28	2.04	89.76	93.97
		**Probability**				
	CVC	<0.001	<0.001	<0.001	<0.001	<0.001
	Enzyme	0.983	0.467	0.489	0.439	0.415
	Enzyme × CCV	0.374	0.549	0.210	0.213	0.219

^1^ n for simple effects of minus CVC × minus Enzyme = 7, plus CVC × minus Enzyme = 7, minus CVC × plus Enzyme = 6, plus CVC × plus Enzyme = 7; ^2^ Standard deviation.

**Table 5:** Main and simple effects of coccidia vaccine challenge (CVC) and exogenous enzyme supplementation on nutrient and energy retention, apparent metabolizable energy (AME), and apparent metabolizable energy corrected for nitrogen (AMEn) in 28-day-old broiler chickens fed corn-soybean meal-based diets (14-day post-challenge) ^1^.

Coccidia Vaccine Challenge	Enzyme	Dry Matter, %	Nitrogen, %	Energy, %	AME	AMEn
		**Means for Main Effect of CVC**				
−		74.1	71.0	77.6	3481	3396
+		73.4	69.8	76.2	3421	3333
		**Means for Main Effect of Enzyme**				
	−	73.3	69.5	76.2	3426	3337
	+	74.2	71.2	77.6	3476	3392
		**CVC × Enzyme**				
−	−	74.3	71.2	77.4	3481	3398
+	−	72.2	67.9	74.9	3370	3277
−	+	73.9	70.8	77.7	3480	3395
+	+	74.5	71.7	77.5	3472	3390
		
	SD ^2^	2.60	3.11	2.42	108.61	116.99
		**Probability**				
	CVC	0.481	0.353	0.178	0.177	0.186
	Enzyme	0.374	0.179	0.155	0.253	0.245
	Enzyme × CCV	0.201	0.097	0.238	0.237	0.220

^1^ n for simple effects of minus CVC × minus Enzyme = 6, plus CVC × minus Enzyme = 7, minus CVC × plus Enzyme = 7, plus CVC × plus Enzyme = 6; ^2^ Standard deviation.

**Table 6:** Main and simple effects of coccidia vaccine challenge (CVC) and exogenous enzyme supplementation on nutrient and energy retention, apparent metabolizable energy (AME), and apparent metabolizable energy corrected for nitrogen (AMEn) in 28-day-old broiler chickens fed wheat-corn-soybean meal-based diets (14-day post-challenge) ^1^.

Coccidia Vaccine Challenge	Enzyme	Dry Matter, %	Nitrogen, %	Energy, %	AME	AMEn
		**Means for Main Effect of CVC**				
−		74.0	67.0	77.5	3414	3329
+		73.9	67.2	76.6	3372	3287
		**Means for Main Effect of Enzyme**				
	−	73.1	65.9	76.3	3362	3275
	+	74.8	68.3	77.8	3423	3341
		**CVC × Enzyme**				
−	−	73.9	66.9	77.3	3407	3281
+	−	72.4	64.9	75.3	3318	2726
−	+	74.2	67.1	77.7	3421	3297
+	+	75.5	69.4	77.8	3426	2650
−		73.9	66.9	77.3	3407	3289
	SD ^2^	3.54	4.49	3.29	144.89	156.13
		**Probability**				
	
	CVC	0.942	0.946	0.470	0.471	0.506
	Enzyme	0.230	0.190	0.273	0.298	0.295
	Enzyme × CCV	0.318	0.228	0.421	0.420	0.402

^1^ n for simple effects of minus CVC × minus Enzyme = 7, plus CVC × minus Enzyme = 6, minus CVC × plus Enzyme = 7, plus CVC × plus Enzyme = 6; ^2^ Standard deviation.

**Table 7:** Main and simple effects of coccidia vaccine challenge (CVC) and exogenous enzyme supplementation on apparent metabolizable energy (AME) and apparent metabolizable energy corrected for nitrogen (AMEn) of the test ingredient (wheat) 7- and 14-day post-challenge.

CVC	Enzyme	Day 21 (7-Day Post-Challenge) ^1^		Day 28 (14-Day Post-Challenge) ^2^	
		AME, kcal/kg	AMEn, kcal/kg	AME, kcal/kg	AMEn, kcal/kg
		**Means for Main Effect of CVC**		**Means for Main Effect of CVC**	
−		3379.8	3296.6	3460.2	3370.3
+		2718.3	2609.6	3391.7	3302.7
		**Means for Main Effect of Enzyme**		**Means for Main Effect of Enzyme**	
	−	3063.4	2972.5	3365.7	3277.2
	+	3034.7	2933.8	3486.1	3395.8
		**CVC × Enzyme**		**CVC × Enzyme**	
−	−	3368.9	3290.4	3437.5	3353.3
−	+	3390.6	3302.9	3482.8	3387.2
+	−	2757.9	2654.5	3294.0	3201.0
+	+	2678.7	2564.6	3489.3	3404.4
					
SD ^3^		100.0	105.0	261.3	281.7
		**Probability**		**Probability**	
CVC		<0.001	<0.001	0.512	0.548
Enzyme		0.465	0.349	0.254	0.296
Diet × Enzyme		0.204	0.219	0.473	0.453

^1^ n for simple effects of minus CVC × minus Enzyme = 7, plus CVC × minus Enzyme = 6, minus CVC × plus Enzyme = 7, plus CVC × plus Enzyme = 7; ^2^ n for simple effects of minus CVC × minus Enzyme = 7, plus CVC × minus Enzyme = 6, minus CVC × plus Enzyme = 7, plus CVC × plus Enzyme = 6. ^3^ Standard deviation.

**Table 8:** Main and simple effects of coccidia vaccine challenge (CVC) and exogenous enzyme supplementation on jejunal viscosity (cP) in 21- (7-day post-challenge) and 28- (14-day post-challenge) day-old broiler chickens fed corn-soybean (CS)- or wheat-corn-soybean meal (WCS)-based diets.

CVC	Enzyme	Day 21 (7-Day Post-Challenge)		Day 28 (14-Day Post-Challenge)	
		CS Viscosity ^1^, cP	WCS Viscosity ^2^, cP	CS Viscosity ^3^, cP	WCS Viscosity ^4^, cP
		**Means for Main Effect of CVC**		**Means for Main Effect of CVC**	
−		2.643	3.094	2.811	3.157
+		2.349	2.597	2.711	2.917
		**Means for Main Effect of Enzyme**		**Means for Main Effect of Enzyme**	
	−	2.559	3.116	2.732	3.300
	+	2.433	2.574	2.789	2.774
		**CVC × Enzyme**		**CVC × Enzyme**	
−	−	2.748	3.464 ^a^	2.802	3.410
−	+	2.537	2.723 ^bc^	2.820	2.904
+	−	2.370	2.768 ^b^	2.663	3.190
+	+	2.328	2.426 ^c^	2.758	2.643
					
SD ^5^		0.104	0.218	0.125	0.206
		**Probability**		**Probability**	
CVC		<0.001	<0.001	0.053	0.007
Enzyme		0.008	<0.001	0.259	<0.001
Diet × Enzyme		0.062	0.027	0.441	0.803

^1^ n for simple effects of minus CVC × minus Enzyme = 6, plus CVC × minus Enzyme = 6, minus CVC × plus Enzyme = 7, plus CVC × plus Enzyme = 6; ^2^ n for simple effects of minus CVC × minus Enzyme = 7, plus CVC × minus Enzyme = 6, minus CVC × plus Enzyme = 7, plus CVC × plus Enzyme = 7; ^3^ n for simple effects of minus CVC × minus Enzyme = 7, plus CVC × minus Enzyme = 6, minus CVC × plus Enzyme = 7, plus CVC × plus Enzyme = 7; ^4^ n for simple effects of minus CVC × minus Enzyme = 7, plus CVC × minus Enzyme = 6, minus CVC × plus Enzyme = 7, plus CVC × plus Enzyme = 6; ^5^ Standard deviation. ^a–c^ Means within a column without a common superscript differ (*p* < 0.05).

**Table 9:** Main and simple effects of coccidia vaccine challenge (CVC) and exogenous enzyme supplementation on ileal villi height (VH), crypt depth (CD), and height-to-crypt-depth ratio (VH:CD) in 21-day old broiler chickens (7-day post-challenge).

CVC	Enzyme	Corn-Soybean Meal-Based Diet ^1^				Wheat-Corn-Soybean Meal-Based Diet ^2^		
		Villi Height, µm	Crypt Depth, µm	VH:CD		Villi Height, µm	Crypt Depth, µm	VH:CD
		**Means for Main Effect of CVC**						
−		755.5	132.0	5.857		772.8	137.8	5.814
+		662.8	178.4	3.901		682.0	173.7	4.025
		**Means for Main Effect of Enzyme**						
	−	708.5	153.5	4.895		707.4	165.0	4.407
	+	709.8	156.9	4.863		747.5	146.5	5.431
		**CVC × Enzyme**						
−	−	745.4	136.4	5.543		749.0	157.1	4.799 ^b^
+	−	671.6	170.5	4.247		665.8	173.0	4.019 ^b^
−	+	765.7	127.6	6.172		796.7	118.5	6.830 ^a^
+	+	654.0	186.2	3.554		698.3	174.4	4.031 ^b^
	SD ^3^	95.25	34.32	0.819		67.05	23.17	0.872
		**Probability**						
	CVC	0.028	0.004	<0.001		0.004	0.001	<0.001
	Enzyme	0.973	0.810	0.925		0.164	0.067	0.010
	Diet × Enzyme	0.634	0.394	0.064		0.788	0.050	0.011

^1^ n for simple effects of minus CVC × minus Enzyme = 7, plus CVC × minus Enzyme = 5, minus CVC × plus Enzyme = 6, plus CVC × plus Enzyme = 6; ^2^ n for simple effects of minus CVC × minus Enzyme = 6, plus CVC × minus Enzyme = 7, minus CVC × plus Enzyme = 7, plus CVC × plus Enzyme = 6; ^3^ Standard deviation. ^a,^^b^ Means within a column without a common superscript differ (*p* < 0.05).

## Data Availability

The data presented in this study are available on request from the corresponding author.
